# Increased Ethanol Consumption Following Chronic Psychosocial Stress: Do Oxytocin and Baclofen Hold any Therapeutic Promise?

**DOI:** 10.3389/fpsyt.2013.00148

**Published:** 2013-11-15

**Authors:** Mary R. Lee, Jared W. Bollinger, Lorenzo Leggio

**Affiliations:** ^1^Section on Clinical Psychoneuroendocrinology and Neuropsychopharmacology, Laboratory of Clinical and Translational Studies, National Institute on Alcohol Abuse and Alcoholism, National Institutes of Health, Bethesda, MD, USA; ^2^Intramural Research Program, National Institute on Drug Abuse, National Institutes of Health, Baltimore, MD, USA; ^3^Department of Behavioral and Social Sciences, Center for Alcohol and Addiction Studies, Brown University, Providence, RI, USA

**Keywords:** ethanol consumption, chronic psychosocial stress, stress model, chronic subordinate colony housing, anxiety, oxytocin, baclofen

Both chronic stress (1) and anxiety disorders (2) are risk factors for developing alcohol use disorders (AUDs). Conversely, chronic alcohol consumption can increase anxiety via a rebound effect and acute withdrawal symptoms, thus contributing to anxiety-related development of AUDs and their maintenance (3). As such, there is a rationale and growing interest in developing medications with anti-stress and/or anxiolytic properties as putative treatments for AUDs. To this end, animal models that approximate or are relevant to human conditions of chronic stress and anxiety are relevant to test the effect of potential medications on alcohol-seeking behaviors.

Many rodent models of stress use either conditioned responses (e.g., shocks paired with stimuli) or unconditioned responses (e. g., natural predators) to provoke anxiety (4). Yet these models are of limited relevance to the experience of chronic psychosocial stressors and the resultant anxiety disorders seen in humans. In the study discussed here, Peters and colleagues (5) used a mouse model of chronic psychosocial stress previously developed by the same group (6). The model involves chronic subordinate colony housing (CSC), where a mouse is placed in the same cage with a socially dominant mouse for 19 days. The corresponding control condition is where the mouse is in a single housing (SH) for the same duration. Previous studies with this model, using male mice only, reveal that CSC effects increased anxiety behavior as measured by elevated plus maze and light-dark box (6, 7). Accompanying this anxiety behavior is a reduction body weight gain and thymus weight, increased adrenal weight, and blunted cortisol response to circadian stimulation *in vivo* and adrenocorticotropic hormone (ACTH) challenge *in vitro*. The dysregulation observed in biomarkers of stress response perhaps explains the colonic inflammatory response also observed in this model (6). However, *in vivo*, cortisol levels were elevated in CSC mice compared to SH mice during 5 min exposure to an elevated platform, indicating intact acute stress response (8). Interestingly, exposure to CSC differentially affects hypothalamic expression of vasopressin and oxytocin in the paraventricular nucleus with an increase in the former and no change in the latter (7).

In the present study (5), the authors first validated 14 (vs. the previously validated 19) days of CSC as a stress provocation. Fourteen days of CSC resulted in increased anxiety behavior, as measured by significantly less time spent in the lit box compared to SH mice. Second, they demonstrated that this stressor led to increased ethanol consumption and preference without affecting the same for sweet or aversive non-alcoholic substances, thus suggesting an effect that is specific for alcohol-related behaviors.

The authors also tested the effect of two anxiolytic molecules, baclofen, and oxytocin, on drinking behavior as a consequence of CSC compared to SH. Both baclofen and oxytocin have been proposed as medications for AUDs. Intriguingly, there is preliminary evidence that the anxiolytic, anti-craving action of baclofen might be mediated, perhaps, by an oxytocinergic mechanism in the supraoptic nucleus of the hypothalamus (9). However, in this study the combination of baclofen and oxytocin was not investigated, nor the effects of baclofen on oxytocin levels. Therefore, future studies are warranted to address whether they share common pathways of action.

In preclinical studies, baclofen decreases chronic ethanol consumption in rodents undergoing the alcohol-water “free choice” paradigm (10, 11). Baclofen also reduces lever presses for alcohol in mice and rats (12–16). Perhaps most relevant to study discussed here is that post-dependent rats in a state of withdrawal (which is anxiogenic) after given baclofen, show decreased alcohol self-administration, anxiety behaviors, tremors, and seizures (10, 17–19).

A few human studies show additional promise of baclofen in reducing ethanol consumption and anxiety symptoms. Two double-blind placebo-control studies demonstrated that baclofen is superior to placebo in preventing relapse (20, 21) [but see (22) for conflicting results]. Decreased anxiety levels and biomarkers of the stress response in alcoholic participants are also seen after administration of baclofen (20, 23–25). Furthermore, consistent with the preclinical literature, clinical studies show that baclofen may be effective in reducing alcohol withdrawal symptoms, including withdrawal-related anxiety levels (26, 27).

As for oxytocin, much of the animal research investigating oxytocin within the context of addiction have centered on its ability to inhibit and reverse tolerance to ethanol and morphine (28). Additionally, oxytocin has been shown to decrease alcohol preference (29). In alcohol preferring rats, a single administration oxytocin, i.p. (1 mg/kg) prior to alcohol access, resulted in significantly reduced preference for alcohol lasting 6 weeks (29). To our knowledge, only one small pilot clinical trial in heavy drinkers, has examined the effects of oxytocin on alcohol withdrawal symptoms. Compared with placebo, oxytocin significantly decreased withdrawal symptoms and reduced the amount of lorazepam administered “as needed” during detoxification (30).

In the study by Peters and colleagues, after establishing that 14 days of CSC results in significantly increased anxiety behavior and increased alcohol consumption and preference, this rodent model of stress and anxiety was used to test the effect of baclofen and oxytocin on subsequent drinking behavior. A single dose each of systemic baclofen, oxytocin, and vehicle as well as a single dose of intracerebroventricular (i.c.v.) oxytocin or vehicle was given following 2 weeks of a two bottle free choice period of increasing concentrations of ethanol up to 8%. Ethanol consumption and preference were the main outcome measures, with saccharine and quinine as positive and negative controls, respectively. Baclofen significantly reduced alcohol consumption and preference in both groups of mice. In contrast, oxytocin reduced alcohol consumption only with peripheral administration and only in the SH group. For both drugs, there was a compensatory increase in water consumption, with no significant difference from baseline in total liquid consumption.

A fundamental question, evaluating the results of this study, is the effect of increased alcohol consumption on the anxiogenic phenotype in the CSC group. While the CSC mice exhibit more anxiety behavior and consume more alcohol, the experimental design did not allow for determination of anxiety-like behavior as a consequence of both CSC and alcohol consumption. Therefore, one cannot say whether the stress manipulation together with the exposure to alcohol also resulted in an anxiogenic phenotype. This is important in evaluating the observed effect of both drugs on drinking behavior in the context of each drug’s anxiolytic properties.

It is interesting that baclofen and oxytocin have been shown to decrease alcohol consumption and/or decrease anxiety-related alcohol withdrawal symptoms, both in rodents (10, 28, 29) and in humans (26, 27, 30, 31). However, in this CSC model of anxiety, resulting in induced elevation of alcohol consumption, neither drug was specific for reducing alcohol consumption in the context of stress-induced anxiety.

In preclinical studies using similar paradigms for testing anxiety such as the elevated plus maze, baclofen, when given in doses comparable to those used in this study (3.0 mg/kg), is not anxiolytic. Rather, administration of baclofen results in a non-specific behavioral inhibition (32). Further, in the specific test used in this study to measure anxiety, the light-dark box, while baclofen has not been studied, a GABA_B_ receptor antagonist shows no anxiogenic effect (33). With respect to alcohol, in contrast, in rodent studies, baclofen (1.25 and 2.5 mg/kg) reduces measures of anxiety from alcohol (18, 34). In humans, it decreases the anxiety associated with alcohol withdrawal, as stated above. Considering, therefore, the results of the study by Peters and colleagues, it is possible that the effect of baclofen was to produce a decrease in the rewarding property of alcohol in both housing contexts, that is, the drug’s effect was not specific for the anxiogenic phenotype driving increased alcohol consumption.

The results for oxytocin mirror those seen in another rodent study examining the effect of oxytocin on alcohol consumption outside of a stress context (29). In this study by McGregor and colleagues (29) a single dose of oxytocin (a tenth of that used in the present study: 1 mg/kg) given i.p. reduced alcohol consumption over 6 weeks, without a significant overall change in fluid intake. Notably, Peters and colleagues administered oxytocin both peripherally and centrally, but no effects were seen when it was administered centrally. It is difficult, however, to speculate why central administration did not replicate the same findings of when oxytocin was given i.p. Furthermore, it is unclear why given oxytocin’s well demonstrated anxiolytic effect (35) there was no effect of oxytocin on alcohol consumption post stress-induced anxiety? Despite the anxiogenic phenotype produced as a consequence of CSC, the CSC model has not been shown to impact oxytocin expression, at least in the paraventricular nucleus of the hypothalamus (36). There might, though, be other brain regions where oxytocin is upregulated, such as the suproptic nucleus where dendritic release has been shown to impact CNS oxytocin levels (37). Further, in other stress models, such as social defeat, there is an up-regulation of oxytocin receptor mRNA in the lateral septum and medial amygdala (38). If endogenous oxytocin was modulated due to the CSC stressor, then perhaps no effect of a single dose of intraperitoneal or intracerebroventricular oxytocin was apparent due to a ceiling effect of oxytocin resulting from the stressor. Further research into the neural pathways modulated by both oxytocin and baclofen with respect to drinking behavior in this stress model is warranted. In summary, further characterization of this rodent social stress model to investigate anxiety-like behavior after both stress and alcohol exposure would inform the use of this model to test medications that are themselves anxiolytic. Also, alternative experimental designs employing this animal model might include either administration of the medication of interest before the stress manipulation to see if the medication protects against development of anxiety and/or increased alcohol consumption, or after the social stressor but before alcohol exposure.
